# Not Seeing the Forest for the Trees: Size of the Minimum Spanning Trees (MSTs) Forest and Branch Significance in MST-Based Phylogenetic Analysis

**DOI:** 10.1371/journal.pone.0119315

**Published:** 2015-03-23

**Authors:** Andreia Sofia Teixeira, Pedro T. Monteiro, João A Carriço, Mário Ramirez, Alexandre P. Francisco

**Affiliations:** 1 INESC-ID Lisboa, Lisbon, Portugal; 2 Instituto Superior Técnico, Universidade de Lisboa, Lisbon, Portugal; 3 Instituto de Microbiologia, Instituto de Medicina Molecular, Faculdade de Medicina, Universidade de Lisboa, Lisbon, Portugal; University of Georgia, UNITED STATES

## Abstract

Trees, including minimum spanning trees (MSTs), are commonly used in phylogenetic studies. But, for the research community, it may be unclear that the presented tree is just a hypothesis, chosen from among many possible alternatives. In this scenario, it is important to quantify our confidence in both the trees and the branches/edges included in such trees. In this paper, we address this problem for MSTs by introducing a new edge betweenness metric for undirected and weighted graphs. This spanning edge betweenness metric is defined as the fraction of equivalent MSTs where a given edge is present. The metric provides a per edge statistic that is similar to that of the bootstrap approach frequently used in phylogenetics to support the grouping of taxa. We provide methods for the exact computation of this metric based on the well known Kirchhoff’s matrix tree theorem. Moreover, we implement and make available a module for the PHYLOViZ software and evaluate the proposed metric concerning both effectiveness and computational performance. Analysis of trees generated using multilocus sequence typing data (MLST) and the goeBURST algorithm revealed that the space of possible MSTs in real data sets is extremely large. Selection of the edge to be represented using bootstrap could lead to unreliable results since alternative edges are present in the same fraction of equivalent MSTs. The choice of the MST to be presented, results from criteria implemented in the algorithm that must be based in biologically plausible models.

## Introduction

The use of trees for phylogenetic representations started in the middle of the 19th century. One of their most popular uses is Charles Darwin’s sole illustration in “The Origin of Species” [1]. The simplicity of the tree representation makes it still the method of choice today to easily convey the diversification and relationships between species. Many different methods have been proposed to reconstruct phylogenies, mostly concerned with recovering evolutionary relationships over long periods of time [2]. Each algorithm or method used to infer and draw a tree, makes a series of implicit or explicit assumptions that limit the types of trees generated. This variability in the trees generated by different algorithms using the same data, has important repercussions that frequently go unappreciated by those who use them. At shorter timescales and with limited diversity, conditions that are encountered in population genetics and microevolutionary studies of a single species, the assumptions made by these methods may not be equally valid [3] and a number of other methods have been used when analyzing this data.

Minimum Spanning Trees (MSTs) are becoming increasingly used for representing relationships between strains in epidemiological and population studies of bacterial pathogens. Although MST computation is a classical mathematical problem and its application to evolutionary studies was suggested more than a decade ago [3], it was not until recently, with the advent of multilocus sequence typing (MLST) [4] and particularly whole genome sequencing [5, 6], that they gained popularity as an alternative to eBURST [7]. One appeal of MSTs is the simplicity of their assumptions that reflect the concept of minimal evolution. MSTs simply link together the more closely related individuals in the population, generating a single tree representing all individuals. The Steiner trees [8], generated by the more classical methods for phylogenetic inference, place individuals exclusively in branch tips. By allowing individuals to be placed in interior nodes, spanning trees and MSTs in particular, may better convey the peculiarities of short-term intraspecific evolution [3].

It was also recently pointed out that the optimal implementation of the BURST rules in goeBURST, results in a set of disjoint MSTs [9]. These trees group sequence types (STs) that differ by a maximum threshold number of alleles from at least one other ST in the group. These groups or connected components are frequently referred to as clonal complexes (CCs). In fact, goeBURST is a maximum weight problem that together with MSTs are particular cases of graphic matroids [9]. But, as it is well known, MSTs are in general not unique for a given network and this was also recognized in the context of phylogenetic trees [3, 10]. The fact that a single tree is reported from a multitude of possible and equally optimal solutions and that no statistical metrics exist to evaluate them, justified a recent heuristic approach to address these issues [10]. The authors suggested a method based on a mark-recapture approach to estimate the number of possible trees and a bootstrap procedure to evaluate tree credibility.

The problem of counting MSTs has been a challenge for the last decades, namely the development of efficient approaches for counting MSTs in weighted graphs, and different approaches have been described. In 1987, Gavril [11] addressed the problem of counting the number of MSTs by constructing a tree-like recursive structure, the root of which is the subgraph *G* ^′^ formed by removing all non-maximum-weight edges from *G*, and each sub-tree of which is constructed recursively from the components of *G*\*G* ^′^. The minimum spanning trees of *G* can then be counted by multiplying together the numbers of spanning trees at each node of this structure. This method runs in *O*(*nM*(*n*)) time, where *M*(*n*) is the time required to multiply two *n* × *n* matrices. Later, in 1997, Broder and Mayr [12] improved this bound by proposing a method based on a generating function that can be expressed as a simple determinant, where the weights of the edges appear as exponents of polynomials. This method proceeds by factoring the determinant and it works for nonnegative integral edge weights. It runs in *O*(*M*(*n*)) time.

Eppstein [13] took still a different approach and created the concept of equivalent graph. Specifically, one constructs from any given edge-weighted graph *G* an equivalent graph *EG* without weights, with a *sliding transformation*, such that the minimum spanning trees of *G* correspond one-for-one with the spanning trees of *EG*. Having translated the weighted graph to an equivalent unweighted graph, one can compute the number of MSTs by just applying the Kirchhoff’s matrix tree theorem to the new graph.

Note that most of these approaches aim at generating and sampling MSTs, a harder problem than just counting the number of MSTs. Moreover, our approach may be applied to the general case of graphic matroids. As discussed above, the problem of finding an MST is a particular case of graphic matroids [14] and, thus, finding a solution for a given graph consists of solving an instance of graphic matroids [14–16], which can be optimally solved with a greedy approach [17]. One of those greedy approaches is precisely Kruskal’s algorithm [18]. In the general case of graphic matroids, edges may be unweighted, which is usually the case. We just need to define a total order for the edges based on specific criteria, which is precisely what we have in phylogenetic approaches using MSTs [9].

Here, we present an improvement to the mark-recapture/bootstrap approach by introducing a new edge centrality metric taking advantage of determining exactly the number of possible trees and the proportion of the tree space that includes each of the possible edges through an expansion of the Kirchhoff’s matrix tree theorem [19, 20]. Contrary to other methods that depend on edges being weighted, our method just depends on sorting edges in increasing order and, thus, we just require a total order to be defined. This simple approach allowed better performance and can be applied to the general case of graphic matroids.

## Methods

In this section, we describe an exact method to compute the significance of a branch/edge in a given MST representation, and we present a module for the PHYLOViZ software [21] implementing the proposed metric. We start with the formalization of the problem under study and the proposed metric. Then, we show how the metric can be effectively computed.

### Spanning edge betweenness

Let *G* = (*V, E*) be a connected, undirected and weighted graph, with weight function *w*: *E* → *IR*, where *V* is the set of vertices and *E* ⊂ *V* × *V* is the set of edges. A Minimum Spanning Tree (MST) *T* = (*V, E* ^′^) is a subgraph of *G* that is a tree and contains all the vertices of *G*, i.e., that spans over all vertices in *V*, with ∣*E* ^′^∣ = ∣*V*∣−1, and such that ∑_*e*∈*E*^′^_ *w*(*e*) is minimum among all possible spanning trees. It is clear that we can have more than one MST for a given graph *G* and we would like to count how many MSTs exist in *G*. The solution to this problem is provided by the Kirchhoff’s matrix tree theorem [20] for unweighted graphs and by Eppstein [13] for weighted graphs, where the Kirchhoff’s matrix tree theorem is still used but only after some graph transformations.

However, in this paper we are interested in a slightly different question. Given an edge *e* ∈ *E*, we want to know the fraction *δ*_*G*_(*e*) of MSTs where *e* occurs. The value *δ*_*G*_(*e*) is what we call the *spanning edge betweenness* for *e* and it is formally defined as

$$\begin{array}{cc} \delta_{G}\left(e\right) & =\frac{\tau_{G}\left(e\right)}{\tau_{G}}, \end{array}$$ (1)

where *τ*_*G*_ is the number of different MSTs for *G* and *τ*_*G*_(*e*) is the number of different MSTs for *G* where *e* occurs. Note that *δ*_*G*_(*e*) may be zero whenever an edge *e* is not present in any MST, causing *τ*_*G*_(*e*) to be zero. In what follows we write *δ*(*e*), *τ*(*e*) and *τ* whenever *G* is clear from the context.

It remains to see how to compute, as efficiently as possible, the spanning edge betweenness *τ*_*G*_(*e*) for a given *e* ∈ *E*. In next sections, we show how to compute *τ*_*G*_(*e*) and *δ*_*G*_(*e*) when *G* = (*V, E*) is a connected, undirected and unweighted graph, with *n* = ∣*V*∣ vertices and *m* = ∣*E*∣ edges. Note that in this case the number *τ* of MSTs in *G* is equal to the number of spanning trees in *G* and it can be computed directly from the Kirchhoff’s matrix tree theorem [19]. Then we extend our result to weighted graphs and we discuss implementation details.

### Unweighted graphs

Let *F* ∈ {−1, 0, 1}^*n* × *m*^ be the incidence matrix for *G* such that *F*_*i, e*_ = 1 and *F*_*j, e*_ = −1, for *e* = (*i, j*) ∈ *E* where *i* < *j* without loss of generality. Let us also consider the reduced incidence matrix *F* ^(*i*)^ obtained from *F* by deleting row *i*. Note that rank(*F*) = *n*−1, rank(*F* ^(*i*)^) = *n* − 1, and the determinant for any square submatrix of *F* ^(*i*)^, for any *i*, is either 0, −1, or 1. A more interesting observation due to Kirchhoff is that a submatrix (*n* − 1) × (*n* − 1) of *F* ^(*i*)^, for any *i*, is non-singular if and only if its columns correspond to the edges of a spanning tree.

**Theorem 1 (Kirchhoff [19]).** *The spanning trees of a connected and undirected graph G with n vertices are the non-singular* (*n* − 1) × (*n* − 1) *submatrices of the reduced incidence matrix F* ^(*i*)^, *for any i, and the determinants of the submatrices are all* ±1.

Hence, by using Cauchy-Binet theorem on determinants, the number of spanning trees *τ* is given by the Kirchhoff’s well known formula

$$\begin{array}{c} \tau =\det(L^{\left(i\right)}) \end{array}$$ (2)

$$\begin{array}{cc} & =\sum_{S}\;\det\left(F_{S}^{\left(i\right)}\right)\det\left({F_{S}^{\left(i\right)}}^{⊤}\right) \end{array}$$ (3)

$$\begin{array}{cc} & =\sum_{S}\;\det{\left(F_{S}^{\left(i\right)}\right)}^{2}, \end{array}$$ (4)

where *S* ranges over the subsets of *E* with size *n* − 1, *L* = *FF* ^⊤^ is the Laplacian matrix for *G*, and *L* ^(*i*)^ denotes the matrix obtained from *L* by deleting row and column *i*.

We extend this result to compute *τ*(*e*), for *e* ∈ *E*, as follows.

**Theorem 2.** *Given G* = (*V, E*) *an undirected and connected graph, let e* = (*i, j*) ∈ *E and L* ^(*ij*)^ *denote the matrix obtained from L by deleting rows i and j and columns i and j. Then, det*(*L* ^(*ij*)^) *is the number of spanning trees τ*(*e*) *that contain e*.

*Proof*. As discussed above, the total number of spanning trees is given by det(*L* ^(*i*)^), for any *i*. Let *G* ^′^ be the graph where we remove the edge (*i, j*) and *L* ^′^ be the Laplacian for *G* ^′^. Hence, the total number of spanning trees for *G* ^′^ is given by det(*L* ^^′^(*i*)^), for any *i*, and the number of MSTs that contain (*i, j*) is simply given by det(*L* ^(*i*)^) − det(*L* ^^′^(*i*)^). Let us show that det(*L* ^(*ij*)^) = det(*L* ^(*i*)^) − det(*L* ^^′^(*i*)^) or, equivalently, that det(*L* ^(*i*)^) = det(*L* ^^′^(*i*)^) + det(*L* ^(*ij*)^). We have that *L* ^(*i*)^ = *F* ^(*i*)^ *F* ^(*i*)^^⊤^ and *L* ^(*ij*)^ = *F* ^(*i, j*)^ *F* ^(*i, j*)^^⊤^, where *F* ^(*i, j*)^ is obtained from *F* by removing rows *i* and *j*, and, using Cauchy-Binet’s formula, we can show instead that

$$\begin{array}{cc} \sum_{S}\;\det{\left(F_{S}^{\left(i\right)}\right)}^{2} & =\sum_{S^{\prime }}\;\det{\left({F^{\prime }}_{S^{\prime }}^{\left(i\right)}\right)}^{2}+\sum_{S^{*}}\;\det{\left(F_{S^{*}}^{\left(i,j\right)}\right)}^{2} \end{array}$$ (5)

where *F* ^′^ is the incidence matrix for *G* ^′^, *S* ranges over the subsets of *E* with size *n* − 1, *S* ^′^ ranges over the subsets of *E*\{(*i, j*)} with size *n* − 1, and S* ranges over the subsets of *E* with size *n* − 2. Since *S* ^′^ ranges over the subsets of *E*\{(*i, j*)}, we can replace *F* ^′^ by *F* in previous equation. Note also that

$$\begin{array}{cc} \sum_{S^{*}}\;\det{\left(F_{S^{*}}^{\left(i,j\right)}\right)}^{2} & =\sum_{S^{*}\cup \left\{\left(i,j\right)\right\}}\;\det{\left(F_{S^{*}}^{\left(i,j\right)}\times \pm 1\right)}^{2} \end{array}$$ (6)

$$\begin{array}{cc} & =\sum_{S^{*}\cup \left\{\left(i,j\right)\right\}}\;\det{\left(F_{\left(S^{*}\cup \left\{\left(i,j\right)\right\}\right)}^{\left(i\right)}\right)}^{2} \end{array}$$ (7)

because adding edge (*i, j*) to S* and considering *F* ^(*i*)^ instead of *F* ^(*i, j*)^ just adds a term ±1 to each matrix determinant. Therefore,

$$\begin{array}{ll} \sum_{S}\;\det{\left(F_{S}^{\left(i\right)}\right)}^{2} & =\sum_{S^{\prime }}\;\det{\left(F_{S^{\prime }}^{\left(i\right)}\right)}^{2}\\ & +\sum_{S^{*}\cup \left\{\left(i,j\right)\right\}}\;\det{\left(F_{\left(S^{*}\cup \left\{\left(i,j\right)\right\}\right)}^{\left(i\right)}\right)}^{2} \end{array}$$ (8)

which is an equality as the first term on the right side ranges over all subsets of *E* with size *n* − 1 that do not contain (*i, j*) and the second term ranges over all subsets of *E* with size *n* − 1 that do contain (*i, j*).

Hence, using both results, we can easily compute *δ*(*e*) for any *e* ∈ *E*. Note also that the same is true for multigraphs, graphs that allow multiple edges between the same pair of vertices, as both results above hold with the following changes in the Laplacian matrix *L* [22]: if vertex *i* is adjacent to vertex *j* in *G*, then *L*_*ij*_ is equal to the number of edges between *i* and *j*; when counting the degree of a vertex, all loops are excluded.

### Weighted graphs

Let *G* = (*V, E*) be a connected, undirected and weighted graph, with weight function *w*: *E* → *IR*. We can compute a MST for *G* by using the Kruskal’s algorithm [18]:

1. sort *E* with respect to *w* in increasing order;
2. create a forest *M* where each *u* ∈ *V* is a tree;
3. iterate over *E* in increasing order and, for each (*u, v*) ∈ *E*, if *u* and *v* are in different trees, add (*u, v*) to *M* combining both trees as single tree;
4. return *M*.

Note that we may get different MSTs by changing the order obtained in step 1, where we can exchange positions of edges with the same weight. In particular, since it is well known that the sorted list of edge weights is the same for any MST, changing the order allow us to obtain all different MSTs.

We can take this a step further. Consider the algorithm *SEB* for computing the number of MSTs and the spanning edge betweenness for each edge:

1. sort *E* with respect to *w* in increasing order;
2. let *H* = (*V*, ∅) and *τ*_*G*_ = 1;
3. iterate over *E* in increasing order and, while edges have the same weights, add them to *H*;
4. for each connected component *C* in *H*, compute *τ*_*C*_ using Theorem 1, update *τ*_*G*_ = *τ*_*G*_ × *τ*_*C*_, and, for each edge *e* ∈ *C*, compute *τ*_*C*_(*e*) using Theorem 2 and *δ*_*C*_(*e*) using Equation 1;
5. contract all edges in *H* such that each connected component becomes a single vertex;
6. if *H* has more than one vertex, repeat from step 3, otherwise return *τ*_*G*_.

The algorithm *SEB* works similarly to the Kruskal’s algorithm by iterating over edges in increasing order with respect to *w* and, at each main iteration (steps 3 to 6), it considers sets of edges with the same weight. Let *e* ∈ *E* and let *M* ^′^ be the forest obtained in Kruskal’s algorithm after processing all edges *e* ^′^ ∈ *E* such that *w*(*e* ^′^) < *w*(*e*). Let also *H* be a graph where each tree in *M* ^′^ is a vertex, i.e., where each tree was contracted, and where we add all edges in *E* with weight equal to *w*(*e*). Since for some main iteration of *SEB* algorithm we stop after adding edges *e* ^′^ ∈ *E* such that *w*(*e* ^′^) ≤ *w*(*e*), *H* does not contain edges *e* ^″^ ∈ *E* such that *w*(*e* ^″^) > *w*(*e*). Moreover, since we contracted all edges *e* ^′^ ∈ *E* such that *w*(*e* ^′^) < *w*(*e*), all edges in *H* have the same weight *w*(*e*), and we can treat it as an unweighted graph (or, since *H* may be a multigraph, as an unweighted multigraph). Hence, if we consider the connected component *C* of *H* that contains edge *e*, and by using results in previous section, we are able compute the number *τ*_*C*_ of spanning trees for that component and also the number *τ*_*C*_(*e*) of spanning trees for that component where *e* occurs. The key observations clarified in the following lemmas are that we can use this approach to compute the number of spanning trees in *G* and that *δ*_*G*_(*e*) = *δ*_*C*_(*e*).

**Lemma 1.** *Given G* = (*V, E*) *a connected, undirected and weighted graph, with weight function w*: *E* → *IR, the algorithm* SEB *computes the number of spanning trees in G*.

*Proof*. It is clear that an edge *e* ∈ *E* can only permute with another edge *e* ^′^ ∈ *E* to form a different MST iff *w*(*e*) = *w*(*e* ^′^) and, if a MST *M* contains *e*, adding *e* ^′^ to *M* leads to a cycle. Moreover, that cycle can only contain edges with weight equal to or lower than *w*(*e*), otherwise *M* would not be an MST. If we add all edges with weight *w*(*e*) to *M* and contract all edges with weight lower than *w*(*e*), we obtain the graph *H* and the product of the number of trees in each connected component of *H* is the number of ways we can select edges with weight *w*(*e*) for each MST of *G*. By doing this for each different weight in *G* and then multiplying all values, we obtain the number of MSTs *τ* for *G*.

**Lemma 2.** *Given G* = (*V, E*) *a connected, undirected and weighted graph, with weight function w*: *E* → *IR, an edge e* ∈ *E, H the graph obtained in algorithm* SEB *while processing edges with weight equal to w*(*e*), *and C the connected component of H that contains e, we have that δ_G_*(*e*) = *δ_C_*(*e*).

*Proof*. Since a given edge *e* only has influence on the number of trees for the component *C* of *H* where it occurs, the number of trees for all other components *C* ^′^ in *H*, and in any other graph *H* in remaining algorithm iterations, remains the same. In particular, by inspecting algorithm *SEB*,

$$\begin{array}{c} \tau_{G}=\tau_{C}\prod_{C^{\prime }}\tau_{C^{\prime }} \end{array}$$ (9)

and, by a similar construction,

$$\begin{array}{c} \tau_{G}\left(e\right)=\tau_{C}\left(e\right)\prod_{C^{\prime }}\tau_{C^{\prime }}\left(e\right)=\tau_{C}\left(e\right)\prod_{C^{\prime }}\tau_{C^{\prime }} \end{array}$$ (10)

where the last equality holds because edge *e* ∈ *E* does not occur in any *C* ^′^. Therefore, by Equation 1, it follows that *δ*_*G*_(*e*) = *δ*_*C*_(*e*).

### Implementation in PHYLOViZ

We have implemented our metric as a module for PHYLOViZ [21], available at http://www.phyloviz.net/. Our implementation uses the Colt library (http://acs.lbl.gov/software/colt/) for linear algebra operations, including in particular the computation of matrix determinants. Since we are dealing with relatively large sparse graphs, we use the class SparseDoubleMatrix2D in Colt. We also use a disjoint-set data structure to track connected components similarly to what is common in Kruskal’s algorithm implementations [23].

The time complexity of the proposed approach is dominated by the time required to compute the determinants, since the Kruskal’s algorithm runs in *O*(*m* log *n*) time, for a graph with *n* vertices and *m* edges. Computing the determinant for a *n* × *n* matrix can be done in *O*(*n* ^1.5^) time [24]. Hence, for sparse graphs with *m* = *O*(*n*), this method runs in *O*(*n* ^2.5^) time since we have to compute a determinant for each edge. In practice, it runs faster as connected components are usually much smaller than the original graph.

### A more efficient implementation

Beside the motivation of an implementation of a module to PHYLOViZ application, we also implemented an offline version of the module where we used some extra settings to accelerate its execution and allow to obtain results that are not meant to be shown in PHYLOViZ. In this offline implementation, we used the MTJ library (Matrix Toolkit Java, available at https://github.com/fommil/matrix-toolkits-java/) that is is a high-performance library for developing linear algebra applications. MTJ is based on BLAS (http://www.netlib.org/blas/) and LAPACK (http://www.netlib.org/lapack/) for its dense and structured sparse computations.

With this library, we use the LU decomposition to calculate the determinant. We create an upper triangle dense matrix and then we go through all the elements of the diagonal. Instead of multiplying all the determinants as in the module developed for PHYLOViZ we sum the logarithm of each absolute diagonal value, obtaining instead the logarithm of the determinant.

To improve our running time, we used the Java concurrent library for computing edges statistics in parallel. Since the statistics for each edge can be computed independently, we could parallelize statistics computation in a straightforward manner. Note that for computing statistics for each edge we must compute the determinant for a given matrix and, since these computations are independent, we can compute edge statistics in parallel. Package available at https://bitbucket.org/phyloviz/popsim-analysis.

## Results and discussion

The spanning edge betweenness was applied to nine publicly available MLST databases of important human pathogens: *Burkholderia pseudomallei, Campylobacter jejuni, Enterococcus faecium, Haemophilus influenzae, Neisseria* spp., *Pseudomonas aeruginosa, Streptococcus agalactiae, Staphylococcus aureus*, and *Streptococcus pneumoniae*. These databases were retrieved on June 24th, 2014, from public repositories available in different websites (see Acknowledgments for more details). From all publicly available databases, we considered only those that generated graphs with more than 500 unique STs linked to at least one other ST at the single-locus variant (SLV) level. Analyses were performed both with PHYLOViZ, using a new module publicly available, and with a command line implementation developed to take advantage of high performance numerical libraries and of parallelization in multi-core platforms (see Methods for more details). We determined the goeBURST forest of each species by linking STs at SLV level, double-locus variant (DLV) level and triple-locus variant (TLV) level. Unless otherwise stated, the analyses were performed on the forest generated by creating trees linking STs at the SLV level. Details on how to reproduce this study, including copies of used databases, are also available at https://bitbucket.org/phyloviz/popsim-analysis.

We calculated the number of possible MSTs in the largest CC of each of these species (Table 1). As expected, even only for the largest CC, the number of possible MSTs is quite large, in fact it exceeds a googol [25] for most of the species considered. When MST results are presented, a single tree is usually shown. This tree is chosen from among the space of possible trees, following a set of rules or simply as a consequence of the algorithm used and the input order of the nodes [10]. The goeBURST algorithm implemented in PHYLOViZ, selects the final tree according to a set of well defined rules that guarantee the uniqueness and consistency of the selected tree, independently of the input order of the nodes [9, 21]. The impact of the application of each of the rules on the space of possible trees for the largest CC of each species is presented in Table 2. For most species, a single tree is obtained when applying up to the second tiebreak rule (higher number of DLVs), but in the case of *B. pseudomallei, C. jejuni* and *Neisseria* spp. a single tree is only obtained when invoking rules up to the third tiebreak rule (higher number of TLVs). In the case of *S. pneumoniae* only the last tiebreak rule (higher number of STID) results in a single tree. Large reductions in the available tree space can be seen with the application of each goeBURST rule and this can be used to evaluate the impact of each rule on the final phylogenetic hypothesis proposed by the algorithm.

**Table 1:** Statistics relative to the largest CC linking STs at the SLV level. Columns represent the number of STs, the number of edges, the total number of possible MSTs and the compactness and clustering indexes. The compactness index quantifies how directly connected individuals in the network are. The clustering index quantifies how close the neighbors of a given individual are from a complete graph (clique). Each index is an average after computing the index individually for each ST.

**Data sets**	**Statistics for the largest CC**				
**Species**	**# STs**	**# Edges^a^**	**# MSTs**	**Compactness**	**Clustering**
*B. pseudomallei*	624	1476	10^276.74^	0.008	0.283
*C. jejuni*	2318	9288	10^1440.45^	0.003	0.600
*E. faecium*	610	1906	10^338.32^	0.010	0.464
*H. influenzae*	150	668	10^94.31^	0.059	0.678
*Neisseria* spp.	2011	12701	10^1521.63^	0.006	0.627
*P. aeruginosa*	101	159	10^22.81^	0.031	0.442
*S. agalactiae*	519	2520	10^365.79^	0.019	0.690
*S. aureus*	1089	8317	10^970.83^	0.014	0.796
*S. pneumoniae*	1275	5203	10^788.28^	0.006	0.641

^a^The number of edges, refers to the total number of edges linking all STs that are SLVs of each other

**Table 2:** The effect of goeBURST tiebreaking rules in reducing MST space. Each column represents the number of possible MSTs after each break rule.

**Data sets**	**# Trees in the largest CC^a^**					
**Species**	**All edges^b^**	**SLV**	**DLV**	**TLV**	**Frequency**	**STID**
*B. pseudomallei*	10^276.74^	10^209.93^	10^5.33^	1	1	1
*C. jejuni*	10^1440.45^	10^632.52^	10^2.16^	1	1	1
*E. faecium*	10^338.32^	10^208.72^	1	1	1	1
*H. influenzae*	10^94.31^	10^0.95^	1	1	1	1
*Neisseria* spp.	10^1521.63^	10^390.84^	10^3.06^	1	1	1
*P. aeruginosa*	10^22.81^	10^11.56^	10^0.6^	1	1	1
*S. agalactiae*	10^365.79^	10^42.02^	1	1	1	1
*S. aureus*	10^970.83^	10^48.71^	1	1	1	1
*S. pneumoniae*	10^788.28^	10^209.02^	10^2.64^	10^0.60^	10^0.60^	1

^a^ The CC was determined by linking groups of STs that were SLV of at least another ST in the group.

^b^ The number of edges linking all STs that are SLV of each other in the CC

The magnitude of the reduction of tree space varies between the species considered (Table 2). It is clear that the number of STs influences the number of possible trees, with the number of possible trees increasing with the number of STs (Table 1). But this relationship is complex, with the number of possible edges linking STs at the SLV level having a similar and equally significant influence on tree space. For instance, when comparing the largest CCs of *B. pseudomallei* and *E. faecium*, although both have a similar number of STs, the latter has a higher number of possible edges and trees (Table 1). An even more striking example is the comparison between the largest CCs of *S. aureus* and *S. pneumoniae*, with the former having a smaller number of STs, but a higher number of possible edges and trees (Table 1). The measurements of compactness and clustering of the tree of the largest CC capture properties that may be related to intrinsic characteristics of each species. These measures have several formulations in the literature [26], but for the results presented here, these were defined as follows. Given a graph *G* = (*V, E*), compactness describes how well a vertex *u* is connected in the graph, being defined as the quotient between the vertex degree *d*_*u*_ and the maximum number of possible neighbors ∣*V*∣ − 1, i.e., *d*_*u*_/(∣*V*∣ − 1). The clustering coefficient describes how well connected is the neighborhood of a vertex *v*, being defined as the quotient between the number of edges among neighbors *N*_*v*_ of *v* and the maximum number of possible edges among them, i.e., 2∣{(*v, w*) ∈ *E* ∣ *v, w* ∈ *N*_*u*_}/*d*_*u*_(*d*_*u*_ − 1). The compactness and clustering coefficient of the graph are defined as the average of the vertex compactness and clustering coefficient over vertices in *G*, respectively. These definitions allow us to also compute these values for each connected component, which is of particular interest for the data under analysis. We note also that, although these two measures are related, they allow us to discriminate some interesting graph characteristics, which for the data under analysis may be related to mutation and recombination events. For instance, values of compactness < 0.010 are associated with *B. pseudomallei, C. jejuni, Neisseria* spp. and *S. pneumoniae*; species that also reach higher tiebreak rules to identify a single tree (Table 2). These species are known to have high rates of recombination [27–30]. The existence of recombination can generate STs with multiple possible pathways of descent, which in turn would be expected to affect a graph’s compactness. The goeBURST algorithm in PHYLOViZ can be run by creating sets of disjoint trees linking STs at DLV or TLV level and the result of this analysis for the largest CC of each of the species considered is presented in Table 3. As expected, as we go from SLV to TLV, the higher number of STs and possible edges in the largest CC results in higher numbers of possible trees. The tree space at any given level, when considering the entire forest, is the product of the number of trees for each CC and is greatly influenced by the largest CC, hence our decision to present the analysis of the largest CC only for simplicity.

**Table 3:** Statistics relative to the largest CC linking STs at the SLV, DLV and TLV levels of construction. SLV means that the graph contains only edges linking STs that are SLVs, DLV means that edges were drawn between STs that are SLVs or DLVs of each other, and TLV that edges were drawn between STs that are SLVs, DLVs or TLVs of each other, according to the rules implemented in goeBURST.

**Data sets**	**Statistics for the largest CC**								
**Species**	**SLV**			**DLV**			**TLV**		
	**#STs**	**# Edges**	**#MSTs**	**#STs**	**# Edges**	**#MSTs**	**#STs**	**# Edges**	**#MSTs**
*B. pseudomallei*	624	1476	10^264.87^	979	12490	10^476.77^	1055	59337	10^534.44^
*C. jejuni*	2318	9288	10^1361.82^	3693	109700	10^2278.37^	6668	589783	10^3773.19^
*E. faecium*	610	1906	10^338.32^	734	15199	10^406.23^	889	56221	10^470.40^
*H. influenzae*	150	668	10^94.31^	175	1086	10^56.66^	576	7323	10^199.37^
*Neisseria spp.*	2011	12701	10^1521.63^	8085	375483	10^5612.23^	9919	1174806	10^6512.54^
*P. aeruginosa*	101	159	10^9.50^	927	3644	10^249.99^	1544	18588	10^517.10^
*S. agalactiae*	519	2520	10^336.78^	680	19699	10^486.93^	681	40310	10^488.63^
*S. aureus*	1089	8317	10^970.83^	2079	100394	10^1747.89^	2463	193822	10^1955.66^
*S. pneumoniae*	1275	5203	10^788.28^	8048	161986	10^4190.83^	9536	453859	10^4904.35^

**Fig 1:**
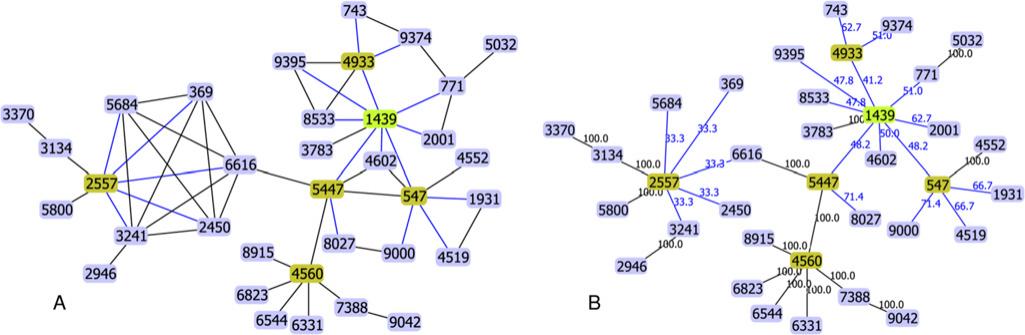
Representation of *S. pneumoniae* CC1439 using PHYLOViZ. A) Representation of all edges linking STs at SLV level. B) Representation of edges from the MST selected after application of the goeBURST rules, with the spanning edge betweenness for each edge.

We have previously proposed that the tiebreak rule reached before deciding if an edge should be drawn, could be used to evaluate the reliability of the represented hypothetical pattern of descent [9]. The spanning edge betweenness can be used for the same purpose, with results that are similar to those of the bootstrap procedure used frequently to support the grouping of taxa on trees [2]. In Fig. 1A we represent all possible edges that could be drawn between STs differing at a single locus (SLVs) in CC1439 of *S. pneumoniae*. While several STs are only linked by one possible edge to another ST, others are linked by several edges to a number of different STs of which they are SLVs. The goeBURST algorithm will then choose which edges should be represented in the final tree from among the edges found in the 88,833,024 possible MSTs of CC1439. In Fig. 1B is represented the MST identified using the goeBURST rules. On each edge is also indicated the percentage of the equivalent MSTs where that edge is found (the spanning edge betweenness). As expected, all the edges that were unique in Fig. 1A were found in all equivalent MSTs, such as the edge between ST6544 and ST4560. On the other hand, the represented edges of STs that could be linked by multiple possible edges, such as ST369, are present in a lower number of possible trees (in this case 33.3% or 29,611,008 trees). With a given set of STs, a higher proportion of equivalent MSTs including the represented edge, means that fewer alternatives are possible and this can be interpreted as a higher confidence in the represented edge.

Another important conclusion that can be drawn from calculating spanning edge betweenness is that all alternative edges linking a given ST to other STs are present in the same proportion of equivalent MSTs. For instance, ST1931 is linked by two possible edges, either to ST4519 or to ST547 (Fig. 1A), and both edges are represented in 66.7% of all equivalent MSTs (see the chosen edge by goeBURST in Fig. 1B). This means that the mark-recapture/bootstrap approach suggested previously to choose between alternative edges [10], cannot provide an adequate solution to the problem of selecting the most adequate edge. In the space of MSTs, alternative edges are equally represented and so any dominance of a given edge in the mark-recapture procedure will be a consequence of the limitations of the procedure and should not be used as selection criterion. The choice between alternative edges must be based on well defined criteria that should reflect an underlying model of microbial evolution. The goeBURST rules have such an underlying model [7, 9, 31] and offer a robust method to select a tree from a forest of MSTs.

**Fig 2:**
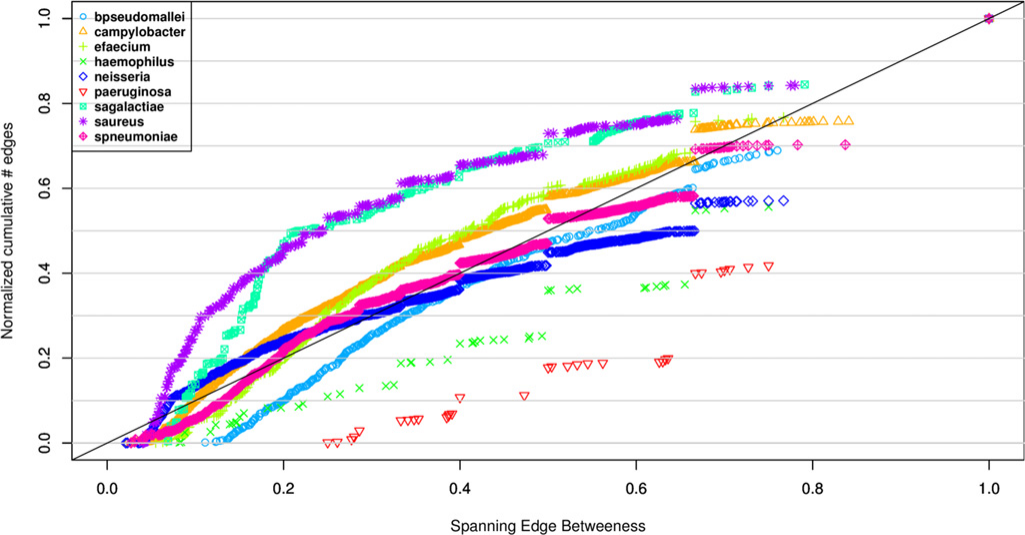
Cumulative spanning edge betweenness of the forest of MSTs at the SLV level selected by goeBURST for the different bacterial species. The fraction of MSTs where a given edge is present is computed for each edge, considering all CCs for each bacterial species. The plot is performed cumulatively and the number of edges normalized (for values between 0 and 1). The diagonal represents a putative case where each value of spanning edge betweenness is represented by the same number of edges.

Fig. 2 and Supplementary Figures show the cumulative distribution of the spanning edge betweenness of all edges, in the forest of all CCs, calculated at the SLV level by the goeBURST algorithm in PHYLOViZ. The distribution of spanning edge betweenness of the edges of the MSTs selected by goeBURST is variable between species. In contrast to the number of MSTs discussed above, there is not a dominant role of recombination in determining the shape of the distribution, since the species identified previously as being recombinogenic are not homogeneous in their distributions. These differences possibly reflect differences in size of the data set considered, as well as a more complex interplay of the intrinsic properties of each species, such as mutation and recombination rates and possibly their ratio.

Centrality measures are important in a large number of graph applications, from search and ranking to social and biological network analysis [26]. Most of these measures are calculated upon the nodes/vertices. With node centrality we can measure the relative importance of nodes within a graph [32] but our interest can be to study the importance of links/edges on a network. A first approach was done by Girvan and Newman [33] where they defined edge betweenness, generalizing Freeman’s betweenness centrality [34] to edges, as the number of shortest paths between pairs of vertices that run along an edge, with a direct application on the identification of community structures in networks. There are, however, other problems where alternative definitions of edge centrality are required, as is the case with the statistical evaluation of phylogenetic trees.

Here, we present a new edge centrality metric, the spanning edge betweenness, defined as the fraction of MSTs containing a given edge. We provide the required results and methods to exactly compute this metric. Since we rely on the Kirchhoff’s matrix tree theorem, thus needing to compute several determinants for slightly different matrices, we plan to investigate how to accelerate these computations by reusing previous computations and by using more efficient methods for sparse positive semi-definite matrices decomposition, such as those based on Cholesky’s decomposition [35]. Currently, our solution allows the algorithm to run on a common laptop in reasonable time (Table 4). For data sets with few STs, such as *H. influenzae* and *P. aeruginosa* (Table 1), it takes less than 2 seconds. However, for data sets with a larger number of STs, such as *C. jejuni*, it can take almost one hour. The running time will depend, mostly, on the number of STs of each data set, that is clearly related to the dimensions of the matrix representing the relationships between STs. Hence, the number of STs is directly related to the number of operations required to calculate determinants.

**Table 4:** Time to compute the number of MSTs in all CCs with STs linked at SLV level. Time presented in seconds, using an Intel i7 a 2.3GHz, with 6GB of RAM.

**Data sets**	**Runtime (s)**
*B. pseudomallei*	16.8
*C. jejuni*	2759.3
*E. faecium*	23.2
*H. influenzae*	1.6
*Neisseria* spp.	1489.9
*P. aeruginosa*	2.0
*S. agalactiae*	13.1
*S. aureus*	277.2
*S. pneumoniae*	362.5

The comparison between this metric and other well known centrality metrics should also be investigated in the context of complex network analysis, as it provides a rather different approach for evaluating edge relevance or significance. The analyses of MLST data sets available in public databases show the usefulness of spanning edge betweenness in evaluating MSTs as proposals for phylogenetic relationships, and in providing confidence levels for each selected edge in the final tree. These analyses also highlight the impossibility of selecting an MST based on the statistical support of the edges, and reinforce the importance of the biological plausibility of the model underlying the criteria for edge selection in presenting the best possible MST-based-proposal for the phylogenetic relationship of the entities under analysis. The use of bootstrap values became a key method to assess nodal support in phylogenetic trees [2]. The spanning edge betweenness proposed here offers a similar tool for the evaluation of MSTs in phylogenetic studies.

## Supporting Information

S1 Fig Cumulative spanning edge betweenness of the forest of MSTs at the SLV level selected by goeBURST for *Burkholderia pseudomallei*. The fraction of MSTs where a given edge is present is computed for each edge, considering all CCs. The plot is performed cumulatively and the number of edges normalized (for values between 0 and 1). The diagonal represents a putative case where each value of spanning edge betweenness is represented by the same number of edges.
(TIF)

S2 Fig Cumulative spanning edge betweenness of the forest of MSTs at the SLV level selected by goeBURST for *Campylobacter jejuni*. The fraction of MSTs where a given edge is present is computed for each edge, considering all CCs. The plot is performed cumulatively and the number of edges normalized (for values between 0 and 1). The diagonal represents a putative case where each value of spanning edge betweenness is represented by the same number of edges.
(TIF)

S3 Fig Cumulative spanning edge betweenness of the forest of MSTs at the SLV level selected by goeBURST for *Enterococcus faecium*. The fraction of MSTs where a given edge is present is computed for each edge, considering all CCs. The plot is performed cumulatively and the number of edges normalized (for values between 0 and 1). The diagonal represents a putative case where each value of spanning edge betweenness is represented by the same number of edges.
(TIF)

S4 Fig Cumulative spanning edge betweenness of the forest of MSTs at the SLV level selected by goeBURST for *Haemophilus influenzae*. The fraction of MSTs where a given edge is present is computed for each edge, considering all CCs. The plot is performed cumulatively and the number of edges normalized (for values between 0 and 1). The diagonal represents a putative case where each value of spanning edge betweenness is represented by the same number of edges.
(TIF)

S5 Fig Cumulative spanning edge betweenness of the forest of MSTs at the SLV level selected by goeBURST for *Neisseria* spp.. The fraction of MSTs where a given edge is present is computed for each edge, considering all CCs. The plot is performed cumulatively and the number of edges normalized (for values between 0 and 1). The diagonal represents a putative case where each value of spanning edge betweenness is represented by the same number of edges.
(TIF)

S6 Fig Cumulative spanning edge betweenness of the forest of MSTs at the SLV level selected by goeBURST for *Pseudomonas aeruginosa*. The fraction of MSTs where a given edge is present is computed for each edge, considering all CCs. The plot is performed cumulatively and the number of edges normalized (for values between 0 and 1). The diagonal represents a putative case where each value of spanning edge betweenness is represented by the same number of edges.
(TIF)

S7 Fig Cumulative spanning edge betweenness of the forest of MSTs at the SLV level selected by goeBURST for *Streptococcus agalactiae*. The fraction of MSTs where a given edge is present is computed for each edge, considering all CCs. The plot is performed cumulatively and the number of edges normalized (for values between 0 and 1). The diagonal represents a putative case where each value of spanning edge betweenness is represented by the same number of edges.
(TIF)

S8 Fig Cumulative spanning edge betweenness of the forest of MSTs at the SLV level selected by goeBURST for *Staphylococcus aureus*. The fraction of MSTs where a given edge is present is computed for each edge, considering all CCs. The plot is performed cumulatively and the number of edges normalized (for values between 0 and 1). The diagonal represents a putative case where each value of spanning edge betweenness is represented by the same number of edges.
(TIF)

S9 Fig Cumulative spanning edge betweenness of the forest of MSTs at the SLV level selected by goeBURST for *Streptococcus pneumoniae*. The fraction of MSTs where a given edge is present is computed for each edge, considering all CCs. The plot is performed cumulatively and the number of edges normalized (for values between 0 and 1). The diagonal represents a putative case where each value of spanning edge betweenness is represented by the same number of edges.
(TIF)
