# Supplementary figures and images for: Not Seeing the Forest for the Trees: Size of the Minimum Spanning Trees (MSTs) Forest and Branch Significance in MST-Based Phylogenetic Analysis
Source: PLoS One. 2015 Mar 23;10(3):e0119315. doi: 10.1371/journal.pone.0119315 (PMC4370493; doi:10.1371/journal.pone.0119315)

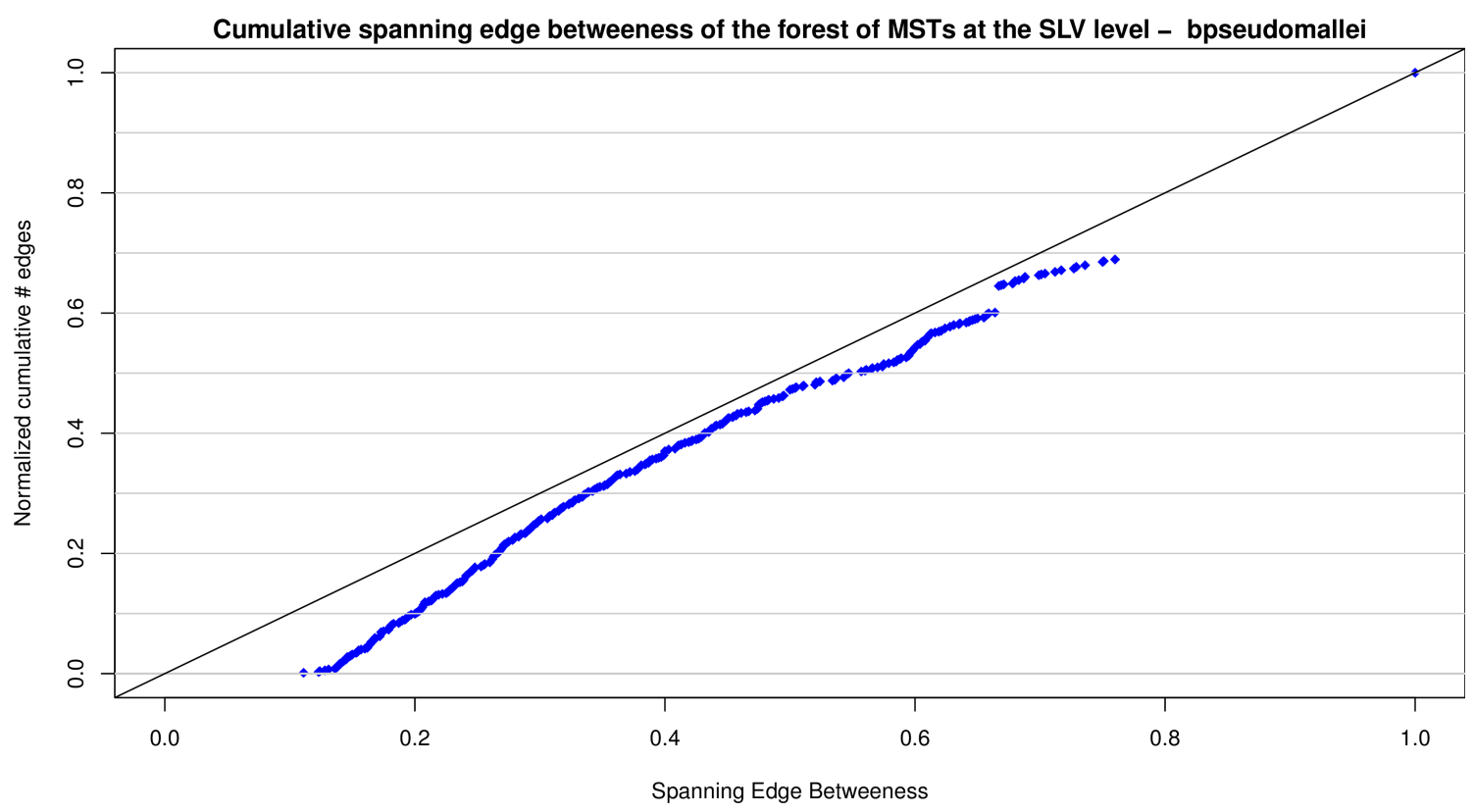

Supplement: S1 Fig — The fraction of MSTs where a given edge is present is computed for each edge, considering all CCs. The plot is performed cumulatively and the number of edges normalized (for values between 0 and 1). The diagonal represents a putative case where each value of spanning edge betweenness is represented by the same number of edges. (TIF) [file pone.0119315.s001.tif]

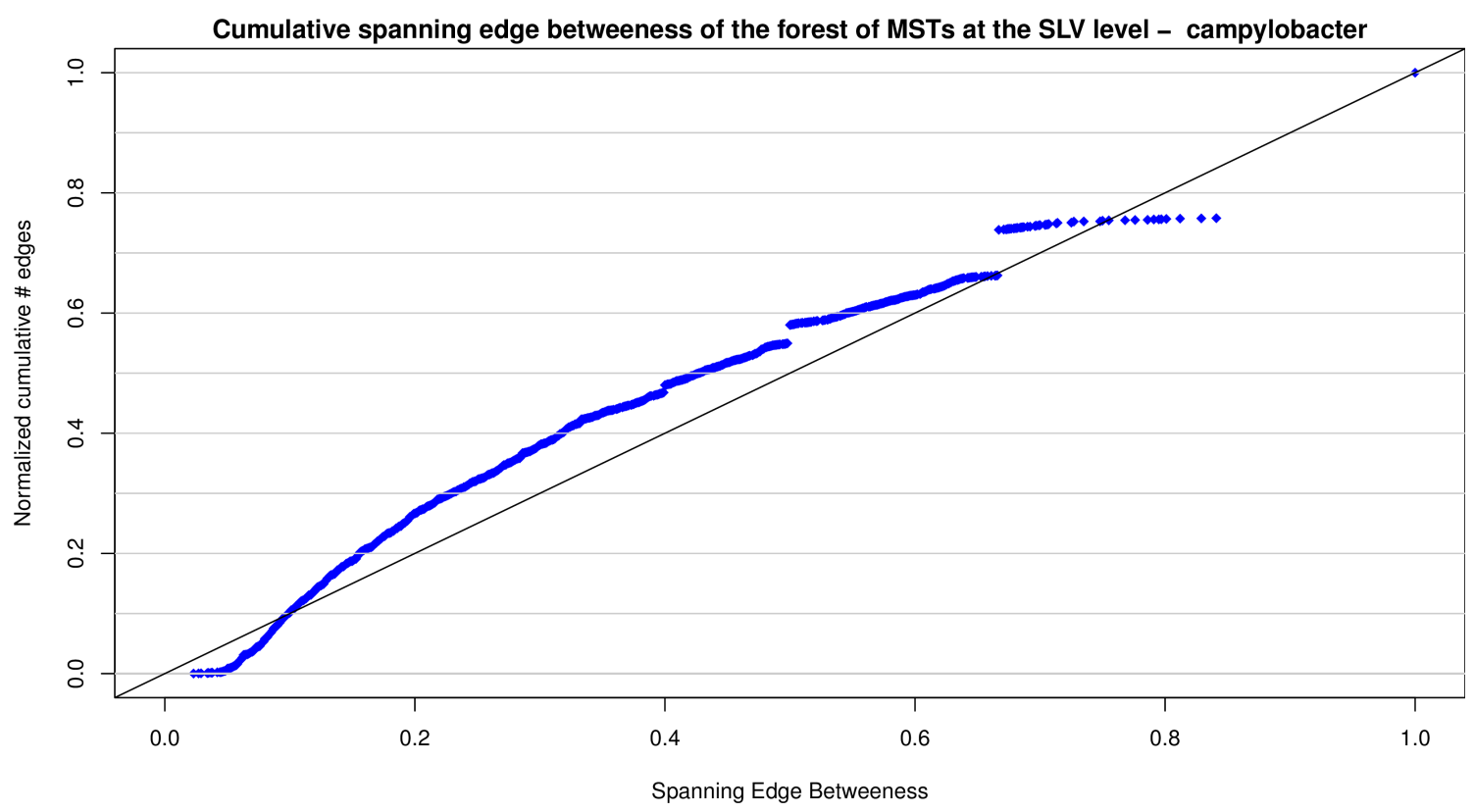

Supplement: S2 Fig — The fraction of MSTs where a given edge is present is computed for each edge, considering all CCs. The plot is performed cumulatively and the number of edges normalized (for values between 0 and 1). The diagonal represents a putative case where each value of spanning edge betweenness is represented by the same number of edges. (TIF) [file pone.0119315.s002.tif]

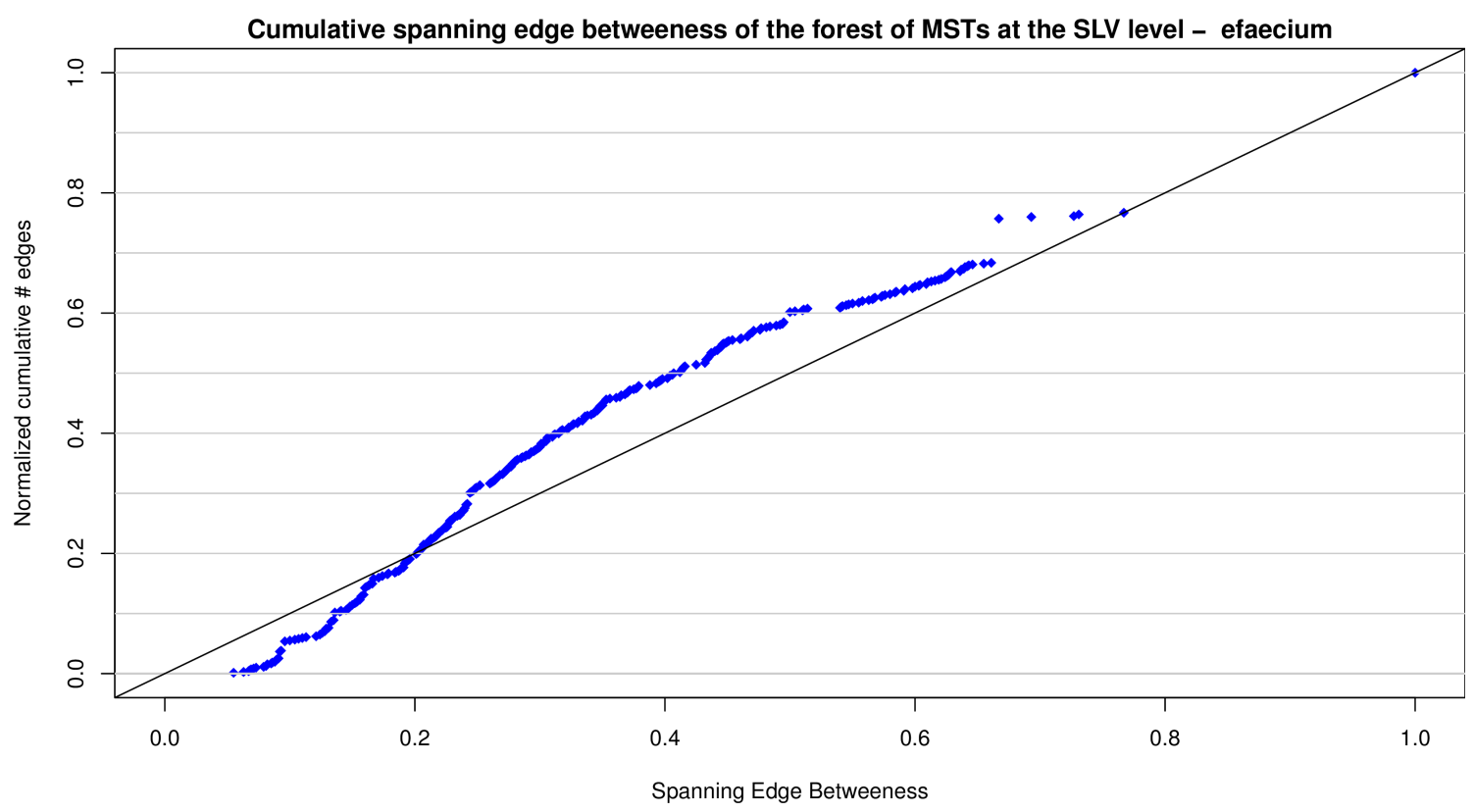

Supplement: S3 Fig — The fraction of MSTs where a given edge is present is computed for each edge, considering all CCs. The plot is performed cumulatively and the number of edges normalized (for values between 0 and 1). The diagonal represents a putative case where each value of spanning edge betweenness is represented by the same number of edges. (TIF) [file pone.0119315.s003.tif]

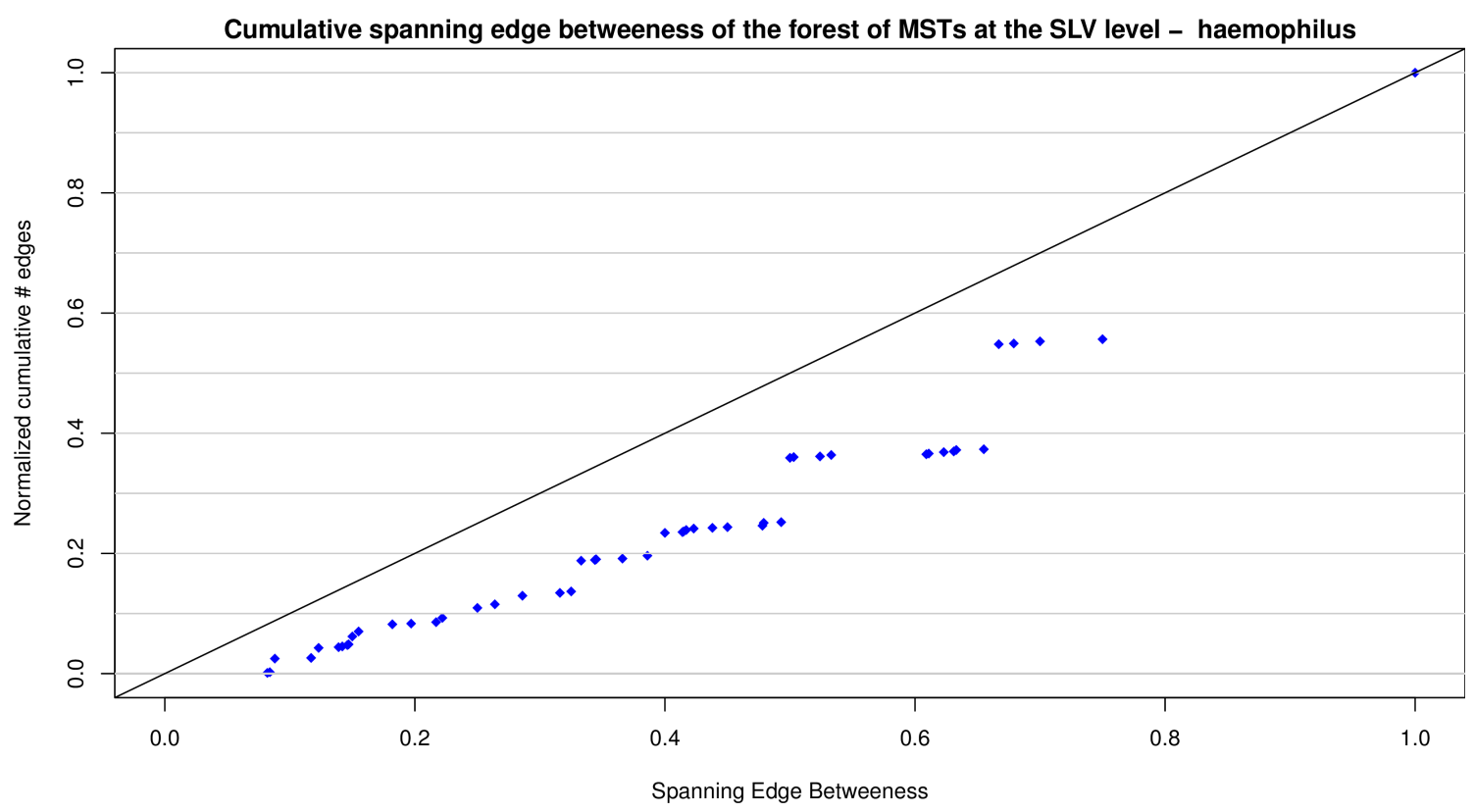

Supplement: S4 Fig — The fraction of MSTs where a given edge is present is computed for each edge, considering all CCs. The plot is performed cumulatively and the number of edges normalized (for values between 0 and 1). The diagonal represents a putative case where each value of spanning edge betweenness is represented by the same number of edges. (TIF) [file pone.0119315.s004.tif]

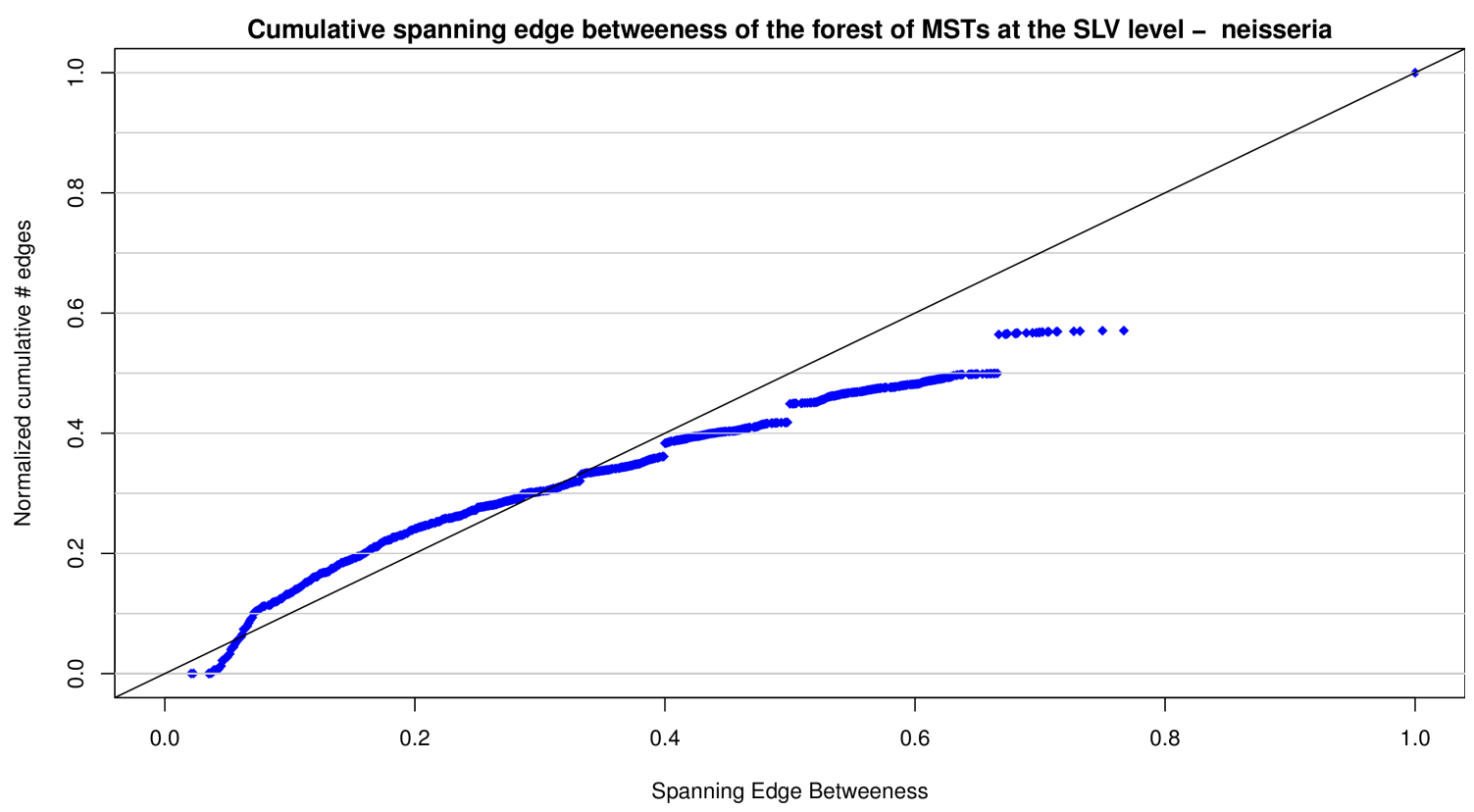

Supplement: S5 Fig — The fraction of MSTs where a given edge is present is computed for each edge, considering all CCs. The plot is performed cumulatively and the number of edges normalized (for values between 0 and 1). The diagonal represents a putative case where each value of spanning edge betweenness is represented by the same number of edges. (TIF) [file pone.0119315.s005.tif]

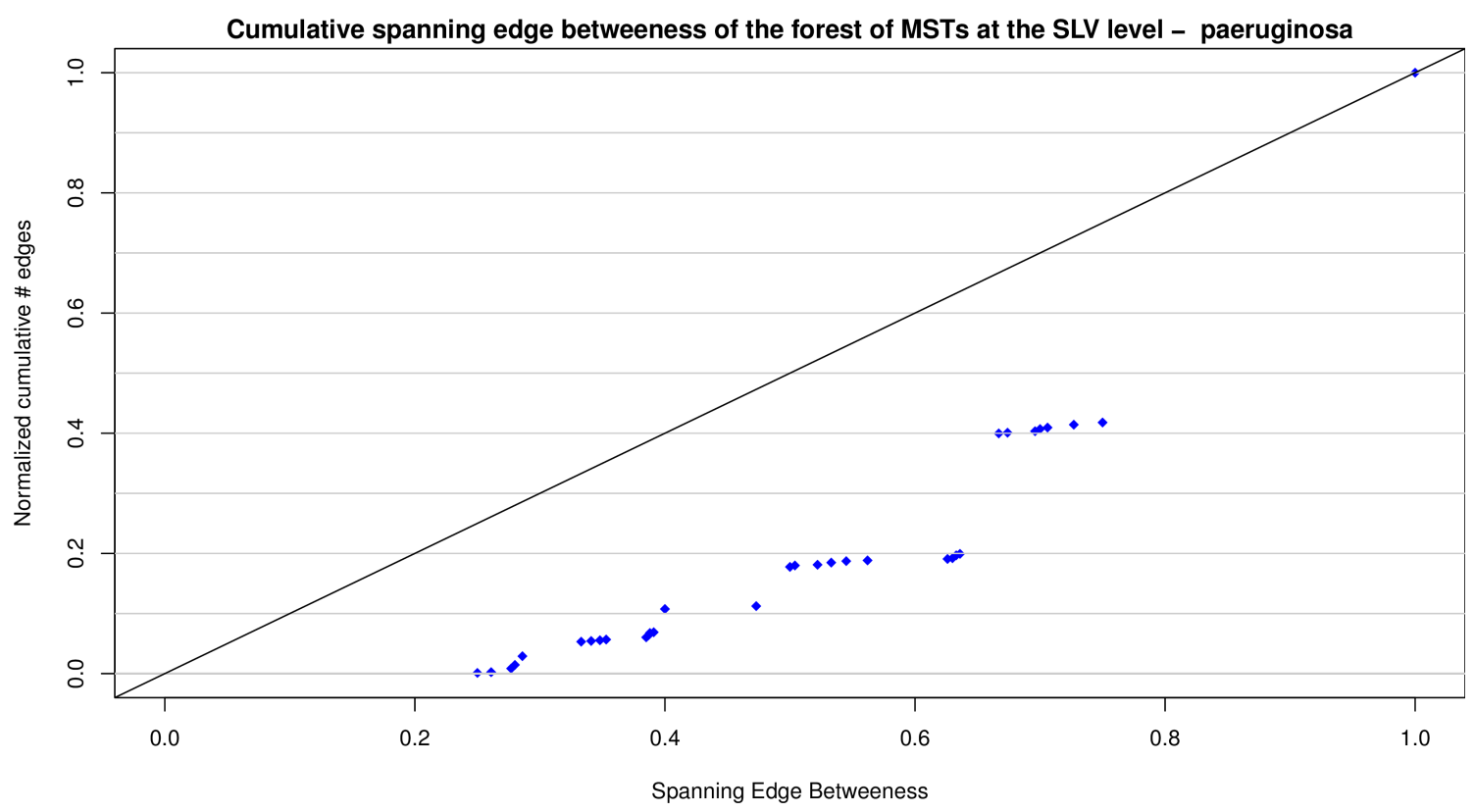

Supplement: S6 Fig — The fraction of MSTs where a given edge is present is computed for each edge, considering all CCs. The plot is performed cumulatively and the number of edges normalized (for values between 0 and 1). The diagonal represents a putative case where each value of spanning edge betweenness is represented by the same number of edges. (TIF) [file pone.0119315.s006.tif]

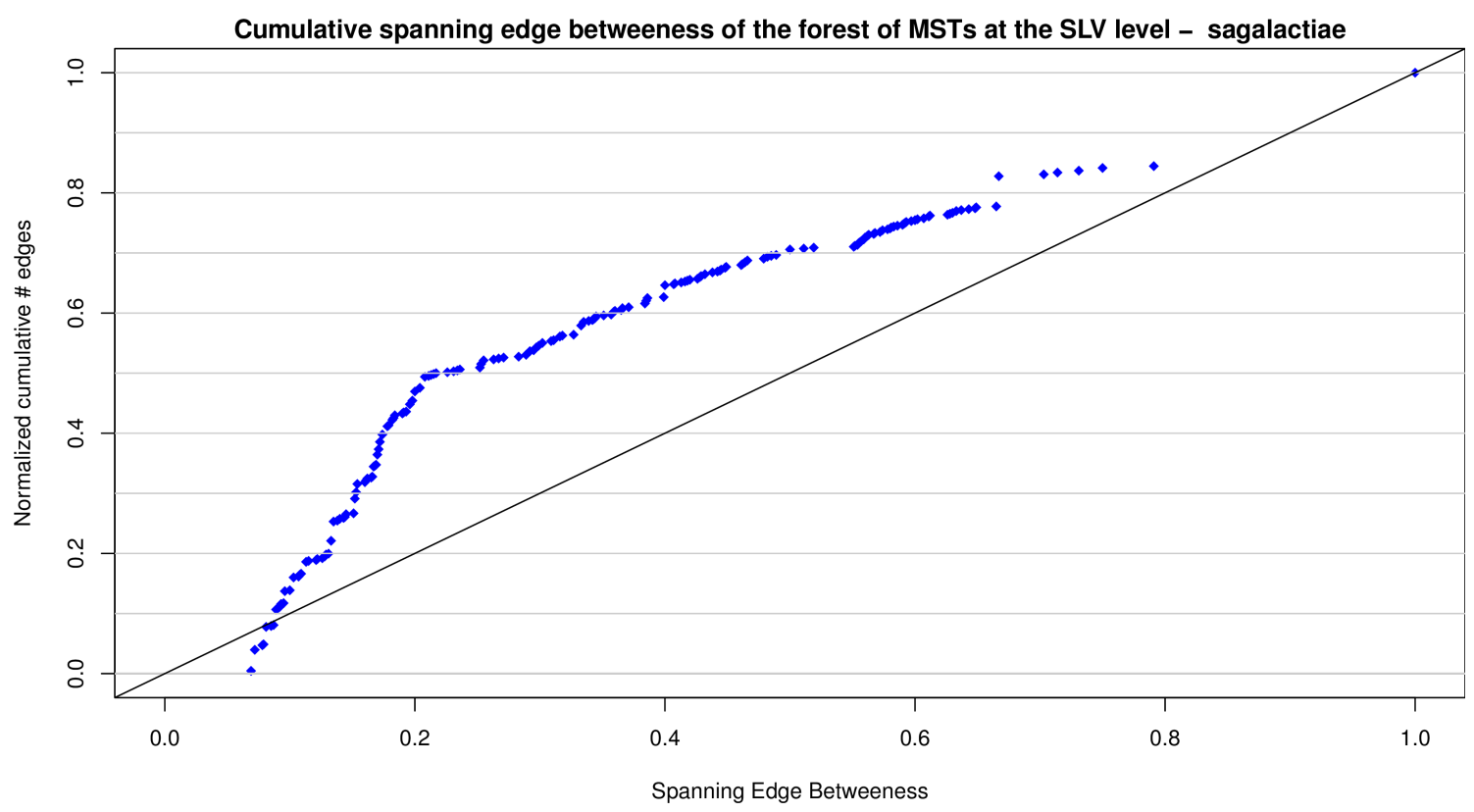

Supplement: S7 Fig — The fraction of MSTs where a given edge is present is computed for each edge, considering all CCs. The plot is performed cumulatively and the number of edges normalized (for values between 0 and 1). The diagonal represents a putative case where each value of spanning edge betweenness is represented by the same number of edges. (TIF) [file pone.0119315.s007.tif]

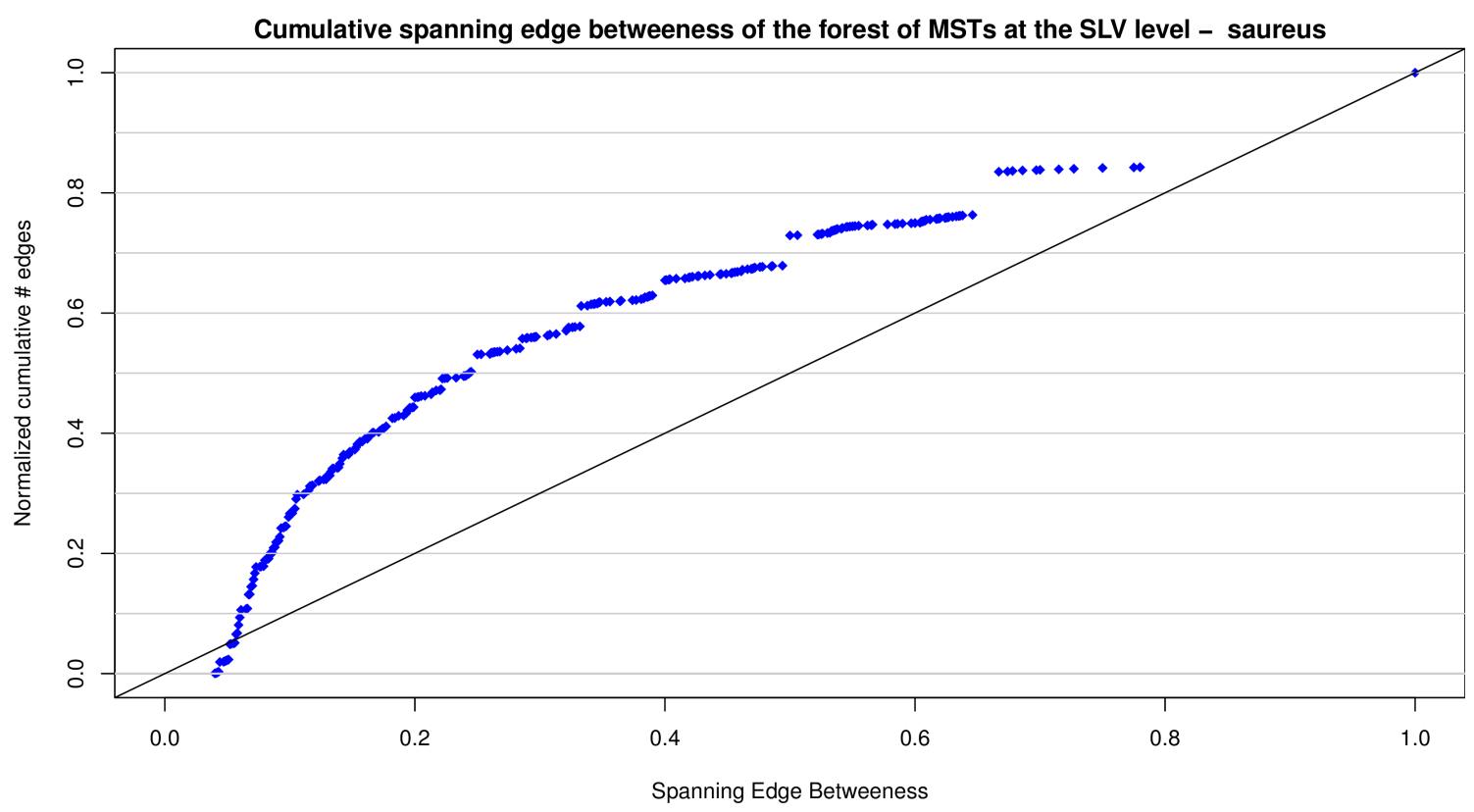

Supplement: S8 Fig — The fraction of MSTs where a given edge is present is computed for each edge, considering all CCs. The plot is performed cumulatively and the number of edges normalized (for values between 0 and 1). The diagonal represents a putative case where each value of spanning edge betweenness is represented by the same number of edges. (TIF) [file pone.0119315.s008.tif]

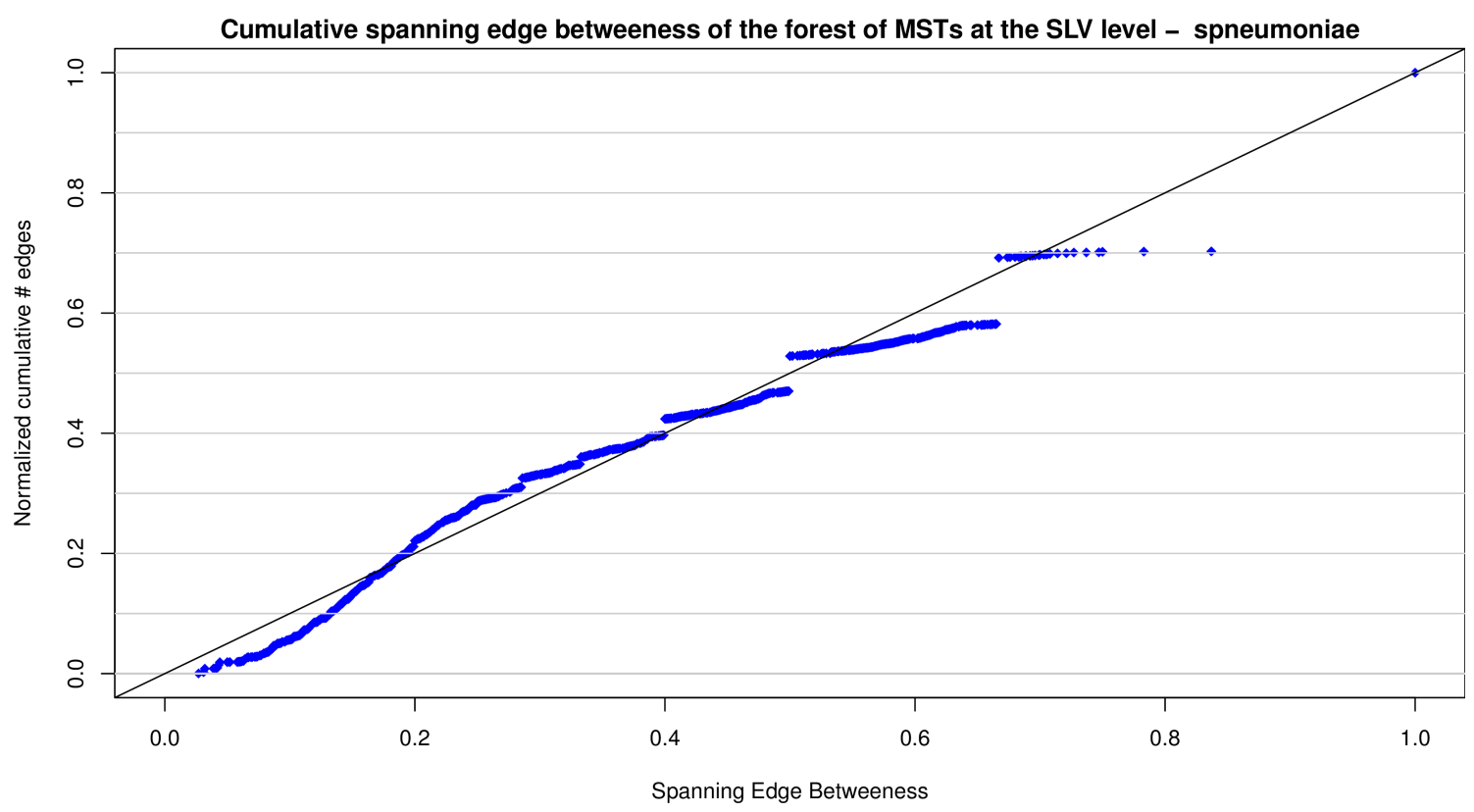

Supplement: S9 Fig — The fraction of MSTs where a given edge is present is computed for each edge, considering all CCs. The plot is performed cumulatively and the number of edges normalized (for values between 0 and 1). The diagonal represents a putative case where each value of spanning edge betweenness is represented by the same number of edges. (TIF) [file pone.0119315.s009.tif]
